# Diaphragmatic pacing after cervical spinal cord injury due to gunshot wound: a 14-year institutional experience

**DOI:** 10.1007/s00701-025-06678-2

**Published:** 2025-09-23

**Authors:** Collin M. Labak, Eric Z. Herring, Elliot Crooks, Stanley F. Bazarek, Gabriel A. Smith, Raymond Onders

**Affiliations:** 1 Department of Neurosurgery, University Hospitals Cleveland Medical Center, Cleveland, OH USA; 2 Department of Surgery, University Hospitals Cleveland Medical Center, Cleveland, OH USA

**Keywords:** Spinal cord injury, Quadriplegia, Diaphragmatic pacemaker, Mechanical ventilation

## Abstract

**Purpose:**

High cervical spinal cord injury (SCI) due to gunshot wound (GSW) represents an extremely devastating injury class not only due to quadriplegia, but also the high incidence of chronic mechanical ventilation (MV) due to injury to the spinal nerves that innervate the diaphragm. Diaphragmatic pacemaker (DP) implantation is a potential option to liberate individuals from chronic MV by assisting with diaphragm contraction and therefore improving respiratory function.

**Methods:**

We conducted a retrospective chart review at our institution to identify patients with high cervical SCI due to GSW who underwent DP implantation and had 6 months or more of clinical follow-up.

**Results:**

Fourteen patients were included for chart review. Twelve patients were male; 9 were African American. Twelve had complete (ASIA A) SCI, of whom 7 had an injury at or above C3. Six of 12 (50%) patients whose MV status was documented achieved 4 h and 24 h per day of MV independence. All patients in whom detailed respiratory function data could be attained showed percentage tidal volumes over baseline requirement (PTVOB) greater than 100% (median:151.9%).

**Conclusion:**

Consistent with previously published data, DP implantation for SCI due to GSW seems to have benefit with regard to MV independence and other respiratory metrics. This held true regardless of injury level or whether the DP was implanted during index hospitalization or in a delayed fashion. DP implantation is a viable option to consider in patients with high cervical SCI after GSW in both the acute and chronic setting to grant patients a potential for MV liberation.

## Introduction

Gunshot wounds (GSW) account for 10–15% of all traumatic spinal cord injury (SCI) in the United States [7]. An analysis of the National Trauma Data Bank estimated that 2,187 cervical SCI between the years of 2015 and 2019 are due to GSW [9]. In the case of upper cervical SCI, chronic respiratory failure is a devastating complication due to dysfunction of the upper cervical nerve roots–C3, C4, and C5–which innervate the diaphragm via the phrenic nerve. This can leave patients reliant on mechanical ventilation (MV), which has major implications related to personal healthcare costs, quality of life, risk of frequent respiratory infections, and overall a significantly reduced life expectancy [6, 8].

If the phrenic nerve and associated motor neurons remain intact after injury, an adjunctive therapy that can be utilized to lessen reliance on MV is the placement of a diaphragmatic pacer (DP) [3]. This surgical procedure involves placement of electrodes along the diaphragm that are attached to a pulse generator, which has been described in previous work [8]. While much of the literature on DP has been focused on SCI and critical illness polyneuropathy, DP has been applied to a number of different conditions including central hypoventilation syndrome and amyotrophic lateral sclerosis [1, 8, 11]. Here, we report on our 14-year experience with the implantation of DP in SCI patients due to GSW, including patient characteristics and outcomes, and provide a review of the current literature.

## Methods

### Study design

A retrospective review of all adult patients who received an implanted DP after SCI due to GSW between the years of 2008 and May 2024 at our institution was performed. Patients with less than 6 months of clinical follow-up were excluded.

### Data collection

The variables collected for each patient included demographic data (age, sex, race, ethnicity), clinical presentation (level of SCI, American Spinal Injury Association Injury Scale (AIS) score, whether or not surgical intervention was required to stabilize the spinal column after their GSW), treatment modality information (timing of DP placement after SCI) and clinical outcomes (length of stay (LOS) after placement, ability to breathe without MV for >/= 4 h and </= 24 h, percentage of tidal volume over baseline requirement (PTVOB) at last follow-up, and complications). Data was managed in a protected Microsoft Excel spreadsheet.

### Basal tidal volume requirement & PTVOB

To calculate basal tidal volume (V_t_) requirement, each patient’s ideal body weight (IBW) was calculated. For men, the equation used was 50 + (0.91 × [height (cm) − 152.4]), and for women, it was 45.5 + (0.91 × [height (cm) − 152.4]). The basal V_t_ was assumed to be 7 ml/kg IBW for men, and 6 ml/kg IBW for women. The PTVOB was then calculated as actual V_t_ prior to discharge divided by the calculated V_t_ based upon IBW.

### Statistical analysis

Data reporting and tabulation was performed with Excel (Microsoft Corp.).

## Results

### Baseline characteristics

A total of 14 patients were included in our study. The mean age for this cohort was 28.4 years, and 85.7% of these patients were male. Additionally, a majority were black/African American (64.3%). Of the group, 85.7% of patients suffered complete spinal cord injuries at the level of their gunshot wound (AIS A). Only one patient in whom DP was placed had a documented incomplete SCI from their GSW (AIS B). All injuries sustained were at the C5 spinal level or above. Additional demographic and clinical information is summarized in Table 1.

**Table 1:** Demographic, injury details and respiratory status pre-DP

Variable *(n=14)*		Frequency	Percentage
Gender	Male	12	85.7%
	Female	2	14.3%
Age (years)	28.4 (22.5)		
Race	Black/African American	9	64.3%
	White/Caucasian	5	35.7%
AIS Classification			
	A	12	85.7%
	B	1	7.1%
	undocumented	1	7.1%
Spinal injury level			
	C2	4	28.6%
	C3	3	21.4%
	C4	4	28.6%
	C5	2	14.3%
	undocumented	1	7.1%
MV-dependent prior to DP placement			
	Yes	14	100%
	No	0	0%
Deceased at time of chart review			
	Yes	4	28.6%
	No	10	71.4%

### Respiratory outcomes after DP implantation

Of the 12 patients whose DP utilization and/or MV-dependence status was recorded, 6 patients successfully reached at least 4 h per day of continuous pacer use. The same 6 patients also were able to be paced for 24 h. Of the 6 patients whose V_t_ at last follow-up visit were documented, all of them had PTVOB well above 100% (median: 151.9%; IQR: 137.1%–169.6%) (Table 2).

**Table 2:** Respiratory status post-DP

			Frequency	Percentage
Patients with MV dependence documented			12	85.7%
Patients with actual Vt documented			6	42.9
≥ 4 h/day ventilator independence at last follow-up			6	50.0%
24 h/day ventilator independence at last follow-up			6	50.0%
	Median	IQR	Q1	Q3
PTVOB (%)	151.9%	32.5%	137.1%	169.6%

### Impact of level of injury

Of the 14 patients included in this study, 7 patients (50%) had injuries at or above C3, 6 patients (42.86%) had injuries at levels C4 or below, and one patient had an undocumented level of injury. Median LOS was shorter in patients with injuries at C3 or above (2 days; IQR 1–3 days) than in patients with injuries C4 and below (10 days; IQR 8.25–12.25 days). Of the patients with documented injury levels and MV independence status, 3 (50%) patients with an injury at or above C3, and 3 (50%) of the 4 patients with an injury at C4 or below reached ≥ 4 h/day of continuous pacing. PTVOB between these same groups was similar comparing patients with injuries at or above C3 (*n* = 2; median 153.8%; IQR 145.3%–162.4%) or at levels C4 or below (*n* = 4; median 151.9%; IQR 137.5%–169.3%). Additional level-based respiratory data is summarized in Table 3.

**Table 3:** Clinical and respiratory status post-DP, by level of injury

Injury level			Frequency	Percentage
C3 and above			7	50.0%
C4 and below			6	42.9%
Undocumented			1	7.1%
Length of stay (days post-DP)	Median	IQR	Q1	Q3
C3 and above	2	2	1	3
C4 and below	10	4.25	8.25	12.25
Undocumented	15			
≥ 4 h/day ventilator independence at last follow-up1			Frequency	Percentage
C3 and above [8]			3	50.0%
C4 and below [8]			3	50.0%
24 h/day ventilator independence at last follow-up1			Frequency	Percentage
C3 and above [8]			3	50.0%
C4 and below [8]			3	50.0%
Patients with actual Vt documented			Frequency	
Total			6	
C3 and above			2	
C4 and below			4	
PTVOB (%)	Median	IQR	Q1	Q3
C3 and above	153.84%	17.17%	145.26%	162.43%
C4 and below	151.85%	31.82%	137.51%	169.32%

^1^Eight [1] patients had undocumented injury levels and/or undocumented ventilation independence status and were therefore excluded

### Timing of DP Implantation

Six of the patients in our cohort had their DP implanted at the same hospitalization as their GSW; another 8 patients had their DP placed in a delayed fashion at a median 5 months post-GSW. Those who had their DP placed during index hospitalization had a median LOS of 12 d post-implantation, whereas those who were brought back had a median LOS of 2.5d post-implantation. Two (33%) of the patients who had their DP placed in the same hospitalization had ≥ 24 h/day MV independence at last follow-up whereas 4 (67%) of the patients who had their DP placed in a later hospitalization from their initial injury had ≥ 24 h/day MV independence at last follow-up. The 5 patients who had DP implantation during index GSW hospitalization had a median PTVOB of 165.35% (IQR 138.34–171.01), while the lone patient who had their DP implanted at a later hospitalization had a PTVOB of 136.67% (Table 4).

**Table 4:** Clinical and respiratory status post-DP, by time to DP placement

Timing to DP placement			Frequency	Percentage
During index hospitalization			6	42.86%
Post-injury			8	57.14%
Timing to DP placement	Median	IQR	Q1	Q3
During index hospitalization (days)	8	2.75	7	9.75
Post-injury (months)	5	9	2.5	11.5
LOS (days post-DP)	Median	IQR	Q1	Q3
All patients	5.5	11.25	1.25	12.5
During index hospitalization	12	5	8.75	13.75
Post-injury	2.5	3.5	1	4.5
≥ 4 h/day ventilator independence at last follow-up^1^			Frequency	Percentage
During index hospitalization [8]			2	33.3%
Post-injury [8]			4	66.6%
24 h/day ventilator independence at last follow-up^1^			Frequency	Percentage
During index hospitalization [8]			2	33.3%
Post-injury [8]			4	66.6%
Patients with actual Vt documented			Frequency	
Total			6	
During index hospitalization			5	
Post-injury			1	
PTVOB (%)	Median	IQR	Q1	Q3
During index hospitalization [3]	165.35%	32.67%	138.34%	171.01%
Post-injury [7]	136.67%	-	-	-

^1^Two [9] patients had undocumented injury levels and/or undocumented ventilation independence status and were therefore excluded

## Discussion

Based on recent estimates, GSWs are the third leading cause of SCI in the United States, and are the second leading cause in patients 16 to 30 years old (SCIMS 2022 Annual Report, National Spinal Cord Injury Statistical Center). Of these, approximately one-third are located in the cervical spine [10]. GSWs present a unique clinical problem in the context of neurologic dysfunction as they often result in injuries to multiple systems based on the path of the projectile. Additionally, the projectile is surrounded by a variable zone of blast injury which can lead to a heterogenous injury pattern [12]. Due to these factors, GSW resulting in SCI represents a heterogeneous group of patients with a combination of central and peripheral nervous system dysfunction.

Because respiratory function localizes to the cervical spinal roots C3, C4, and C5, from which the phrenic nerve arises, about 75% of high cervical GSW lead to chronic MV-dependence [2]. MV carries with it known long-term sequelae, including reduced quality of life, increased cost of healthcare, and a lifelong risk of ventilator-associated events including pneumonia and other infections of the upper and lower airway, barotrauma, and premature death [6, 8]. Therefore, any benefit that can be gained from liberalizing SCI patients from MV may have a significant impact on modifying the trajectory of illness in SCI both in the short and long term.

The largest study to date assessing DP for SCI of any etiology is a retrospective study by Onders et al*.* in which 92 patients’ outcomes were reviewed [8]. A primary endpoint to assess the effectiveness of DP implantation post-GSW is the ability to breathe without the assistance of MV for at least 4 continuous hours per day. The authors found that patients achieving 4 continuous hours without the assistance of MV predicted MV-independence for 24 h per day with high specificity, which is the impetus for this time point. In the most recent prospective study on surgical management to achieve MV independence conducted by Kaufman et al*.*, the group looked at DP implantation alone and with either phrenic nerve reconstruction or diaphragm muscle replacement–this group found that 80% of patients achieved partial or complete weaning from MV, with 40% achieving complete MV independence [4]. All 6 patients in our retrospective study who achieved 4 h per day of continuous DP use were also able to achieve 24 h per day of continuous DP use, thus liberating them from MV.

Additionally in our study, the subgroups were roughly balanced with regard to having DP implantation completed during the same hospitalization as their GSW-induced SCI versus having a delayed implantation of DP following discharge from the hospital to a rehabilitation center. Unfortunately, a subgroup analysis is not possible given the small sizes of our groups, however our data does suggest that DP can successfully liberate patients from MV if placed in an immediate or delayed fashion. If the institution can offer this treatment, it seems to be a low-risk option to implant the device early and attempt to liberate the patient from MV early. However, even if the index injury is cared for at an institution that does not offer DP implantation, patients can be referred in a delayed fashion to receive DP implantation as an attempt to reduce chronic MV dependence.

With regard to the level of injury, prior work has demonstrated that the level of injury in all types of cervical SCI is a factor correlated with independence from MV, specifically differentiating between the C3 and C4 levels [5]. Our study did not find a difference between these two populations, which is likely more attributable to our study being underpowered. However, there may be a different injury pattern in GSW to the spinal cord rather than traumatic injuries where the zone of injury far exceeds the level of injury. One important takeaway from our study regardless of this, is that even in cases where the anterior horns of the phrenic nerve are presumed to be disrupted secondary to the GSW (all cases in our study had GSW affecting C3-5), successful DP and MV freedom can still be accomplished in these patients. Importantly, patients with high cervical SCI are frequently considered for early tracheostomy to reduce outcomes related to MV in patient highly likely to be MV dependent [13]. Similarly, DP after high cervical SCI should also be a consideration as the majority of patients with high SCI are ultimately likely to need some form of MV [2].

This study is bound by the limitations of a small, retrospective cohort and lack of a comparison cohort with regard to drawing larger conclusions about the efficacy and safety of DP implantation. Additionally, our study saw substantial missingness of data likely related to difficulty in following up on patients who came to our hospital system solely for their DP placement (and then returned home afterward). However, the results overall support that DP is a reasonable option to consider for GSW-induced high cervical SCI patients to attempt to achieve MV independence, whether considered during index hospitalization in an acute fashion or in a delayed fashion on follow-up. Further work is needed to make providers caring for these patients aware of the possible options available that may lead to the successful liberation of MV.

## Conclusion

GSW resulting in high cervical SCI are likely to result in significant respiratory dysfunction and ultimately require MV dependence. Our study examined the use of DP in high cervical spinal cord injuries secondary to GSW at our institution. Though there are significant limitations related to a small patient cohort, overall our results support that DP is a reasonable option to consider for GSW-induced high cervical SCI patients to attempt to achieve MV independence, whether considered during index hospitalization in an acute fashion or in a delayed fashion on follow-up.

## Data Availability

No datasets were generated or analysed during the current study.

## References

[1] Amirjani N, Kiernan MC, McKenzie DK, Butler JE, Gandevia SC (2012) Is there a case for diaphragm pacing for amyotrophic lateral sclerosis patients? Amyotroph Lateral Scler 13(6):521–527. 10.3109/17482968.2012.673169 22632380 10.3109/17482968.2012.673169

[2] Call MS, Kutcher ME, Izenberg RA, Singh T, Cohen MJ (2011) Spinal cord injury: outcomes of ventilatory weaning and extubation. J Trauma 71(6):1673–1679. 10.1097/TA.0b013e31821e87c2 21768893 10.1097/TA.0b013e31821e87c2

[3] Hazenberg A, Hofker SS, van der Aa JG, Nieuwenhuis JA, Kerstjens HA, Wijkstra PJ (2013) Diafragmapacemaker: alternatief voor chronische beademing [Diaphragm pacemaker: alternative for chronic ventilatory support]. Ned Tijdschr Geneeskd 157(5):A5572 23369820

[4] Kaufman MR, Bauer T, Campbell S, Rossi K, Elkwood A, Jarrahy R (2022) Prospective analysis of a surgical algorithm to achieve ventilator weaning in cervical tetraplegia. J Spinal Cord Med 45(4):531–535. 10.1080/10790268.2020.1829417 33054689 10.1080/10790268.2020.1829417 PMC9246221

[5] Korupolu R, Uhlig-Reche H, Achilike EC, Reeh C, Pedroza C, Stampas A (2022) Factors associated with ventilator weaning success and failure in people with spinal cord injury in an acute inpatient rehabilitation setting: a retrospective study. Top Spinal Cord Inj Rehabil 28(2):129–138. 10.46292/sci21-00062 35521063 10.46292/sci21-00062 PMC9009196

[6] Krause JS, Cao Y, DeVivo MJ, DiPiro ND (2016) Risk and protective factors for cause-specific mortality after spinal cord injury. Arch Phys Med Rehabil 97(10):1669–1678. 10.1016/j.apmr.2016.07.001 27449321 10.1016/j.apmr.2016.07.001

[7] National Spinal Cord Injury Statistical Center (2013) Spinal cord injury facts and figures at a glance. J Spinal Cord Med 36(1):1–2. 10.1179/1079026813Z.000000000136 23433327 10.1179/1079026813Z.000000000136 PMC3555099

[8] Onders RP, Elmo M, Kaplan C, Schilz R, Katirji B, Tinkoff G (2018) Long-term experience with diaphragm pacing for traumatic spinal cord injury: early implantation should be considered. Surgery 164(4):705–711. 10.1016/j.surg.2018.06.050 30195400 10.1016/j.surg.2018.06.050

[9] Sherrod BA, Young JB, Wilkerson CG, Bisson EF, Dailey AT, Mazur MD (2024) Epidemiology of gunshot-related spinal injuries and related risk factors for in-hospital mortality in the United States from 2015–2019: a national trauma data bank analysis. J Neurotrauma 41(9–10):1112–1121. 10.1089/neu.2023.0081 37694721 10.1089/neu.2023.0081

[10] Sidhu GS, Ghag A, Prokuski V, Vaccaro AR, Radcliff KE (2013) Civilian gunshot injuries of the spinal cord: a systematic review of the current literature. Clin Orthop Relat Res 471(12):3945–3955. 10.1007/s11999-013-2901-2 23479233 10.1007/s11999-013-2901-2 PMC3825909

[11] Slattery SM, Perez IA, Ceccherini I et al (2023) Transitional care and clinical management of adolescents, young adults, and suspected new adult patients with congenital central hypoventilation syndrome. Clin Auton Res 33(3):231–249. 10.1007/s10286-022-00908-8 36403185 10.1007/s10286-022-00908-8

[12] Stefanopoulos PK, Filippakis K, Soupiou OT, Pazarakiotis VC (2014) Wound ballistics of firearm-related injuries–part 1: missile characteristics and mechanisms of soft tissue wounding. Int J Oral Maxillofac Surg 43(12):1445–1458. 10.1016/j.ijom.2014.07.013 25128259 10.1016/j.ijom.2014.07.013

[13] Sun L, Feng H, Mei J et al (2023) One-stage tracheostomy during surgery reduced early pulmonary infection and mechanical ventilation length in complete CSCI patients. Front Surg 9:1082428. 10.3389/fsurg.2022.1082428 37007628 10.3389/fsurg.2022.1082428 PMC10063815

